# Awareness, use, attitudes, and implementation challenges of Bayesian dose prediction software in clinical practice: a multi-organizational survey

**DOI:** 10.3389/fphar.2026.1770100

**Published:** 2026-04-28

**Authors:** Sarah S. Alghanem, Kevin J. Downes, Amit Desai, Sean N. Avedissian, Abdelmoneim Awad

**Affiliations:** 1 Department of Pharmacy Practice, College of Pharmacy, Kuwait University, Kuwait City, Kuwait; 2 Department of Pediatrics, The University of Pennsylvania Perelman School of Medicine, Philadelphia, PA, United States; 3 Division of Infectious Diseases, Children’s Hospital of Philadelphia, Philadelphia, PA, United States; 4 Astellas Pharma Global Development, Inc., Northbrook, IL, United States; 5 College of Pharmacy, University of Nebraska Medical Center, Omaha, NE, United States

**Keywords:** Bayesian dose prediction, clinical decision support systems, implementation barriers, medication safety, pharmacokinetics/pharmacodynamics, precision dosing, therapeutic drug monitoring (TDM)

## Abstract

**Background:**

Bayesian dose prediction software supports model-informed precision dosing to improve therapeutic outcomes. This study evaluated clinicians’ awareness, usage, attitudes, and perceived barriers related to Bayesian dosing software.

**Methods:**

A cross-sectional study was conducted using a validated electronic questionnaire distributed through eight international professional organizations. Descriptive statistics, non-parametric tests, and logistic regression analyses were performed using SPSS version 29.

**Results:**

Of 234 respondents, 190 (81.2%) were eligible and completed the survey. Most were from North America (67.4%), pharmacists (76.3%), and worked in academic hospitals (78.4%). Awareness of Bayesian dosing software was high (82.6%) among respondents, yet only 65% reported its use in clinical practice. The most used software included InsightRx (40.2%), DoseMe (22.5%), and MWPharm (18.6%), mainly for vancomycin (94.1%) and aminoglycosides (50%). The overall median attitude score (IQR) was 4.0 (2.0), indicating positive attitudes, whereas the barrier score was 3.0 (2.0), reflecting high implementation challenges. The most frequently reported barriers were prohibitive licensing costs (65%), lack of institutional support for use and maintenance (50.3%), and limited awareness of its use (49.7%). In multivariable analysis, “region” was the sole predictor of awareness with participants from the Middle East significantly less aware (AOR = 0.14; 95% CI: 0.05–0.42; p = 0.002). Software type was significantly associated with professional role, with pharmacists more likely to select commonly used tools (AOR = 7.2; 95% CI: 1.4–17.9; p = 0.003). A non-significant negative correlation was observed between overall attitude and barrier scores (r = −0.49; p = 0.072).

**Conclusion:**

Despite high awareness and positive attitudes toward Bayesian dosing software, its clinical use remains limited, primarily focused on vancomycin and aminoglycosides, mostly restricted to three commercial tools. Targeted interventions are required to address key implementation barriers related to licensing costs, institutional support, and awareness.

## 1 Introduction

Bayesian dose prediction software has emerged as a valuable tool in clinical pharmacotherapy, particularly for individualizing drug dosing. These tools utilize population pharmacokinetic (PK) models and patient-specific data (e.g., age, weight, and drug concentrations) to estimate individualized PK parameters and provide tailored dosing recommendations that align with predefined PK-pharmacodynamic (PD) targets. Advances in computing have enabled the integration of complex pharmacostatistical models into clinically usable software, improving precision in drug therapy. Bayesian dosing tools have demonstrated superiority over traditional therapeutic drug monitoring (TDM) approaches by increasing the probability of achieving target attainment and enhancing clinical outcomes. In antimicrobial therapy, their application has been associated with improved clinical response rates, reduced adverse events, shorter hospital stays, and lower overall treatment costs (Burton et al., 1991; Neely et al., 2018; Sabourenkov and McLeay, 2019; van Lent-Evers et al., 1999; Zhang et al., 2020).

Despite this evidence and the availability of several Bayesian software platforms, including some freely accessible ones, widespread clinical adoption remains limited. This underutilization is notable across institutions and clinical disciplines, suggesting the presence of barriers to implementation. The main challenges hindering the widespread uptake of Bayesian dose prediction tools, whether academic or commercial, are related to technical, organizational, regulatory, and financial aspects (Darwich et al., 2017). Furthermore, while Bayesian forecasting tools have been associated with promising outcomes, their performance in specific patient populations, such as the critically ill, remains poorly explored, raising concerns regarding generalizability and confidence in use across diverse clinical contexts (Chai et al., 2020).

Accumulated evidence highlights the lack of large-scale evaluations on the integration and impact of Bayesian dose prediction software in routine clinical care (Darwich et al., 2017; Kantasiripitak et al., 2020). Furthermore, end-users’ perspectives remain insufficiently explored, particularly with respect to their awareness, usage patterns, attitudes, and perceived barriers to implementation. Although few studies have directly evaluated clinician experiences with commercial Bayesian dose-optimization platforms, some qualitative work has explored broader perceptions of TDM-related decision-support services. Kumar et al. (2019) assessed the perceived usability of three Bayesian software tools among potential users, identifying varying views regarding user-friendliness (Kumar et al., 2019). More detailed insights are offered by Yager et al. (2022), who conducted qualitative interviews exploring clinicians’ interactions with a vancomycin TDM Advisory Service. Reported barriers included social influences (e.g., prescribing hierarchy), uncertainty about service processes, and challenges in accessing dose recommendations, while facilitators centered on beliefs about improved patient outcomes and the accessibility of dosing advice; notably, trust emerged as a new domain influencing uptake. These findings suggest that the successful implementation of TDM-support technologies depends not only on software interface characteristics but also on broader contextual, interpersonal, and organizational factors. Addressing these gaps in existing literature is essential to promoting the broader adoption of precision dosing technologies and optimizing patient outcomes. Accordingly, the current study was designed as a cross-sectional and multi-organizational survey targeting clinicians’ awareness, use, attitudes, and perceived barriers related to Bayesian dose prediction software. The study was endorsed by the International Society of Pharmacometrics and the American College of Clinical Pharmacology (ACCP1). By capturing insights from practicing clinicians, the findings can help inform educational, institutional, and policy-level interventions that support the effective integration of Bayesian tools into daily clinical workflows.

## 2 Methods

### 2.1 Study design and population

This cross-sectional, multi-organizational study was conducted using a validated questionnaire and disseminated across eight professional organizations: the ACCP1, the American College of Clinical Pharmacy, the Clinical Pharmacometrics Special Interest Group, the International Association of Therapeutic Drug Monitoring and Clinical Toxicology, the Society of Infectious Disease Pharmacists, the Saudi Society of Clinical Pharmacy, the Australasian Society of Clinical and Experimental Pharmacologists and Toxicologists, and the Dutch Association of Hospital Pharmacists.

Ethical approval was obtained from the Kuwait Health Sciences Ethical Committee (Reference: VDR/EC-528). The minimum target sample size was calculated to be 360 participants (Raosoft, 2025). This calculation was performed using the Raosoft calculator, based on a 5% margin of error, 95% confidence level, a population size of 5,600 (corresponding to the total membership of the eight participating organizations), and an assumed response distribution of 50%.

### 2.2 Questionnaire development and validation

As no existing validated instrument addressed the study objectives, a survey was developed and validated. Face validity was initially established through expert review by members of the Outreach and Engagement Taskforce of the Clinical Pharmacometrics Special Interest Group, who assessed the appropriateness of the content, language, and structure. Content validity was subsequently assessed by a panel of seven international experts in pharmacometrics from academic, clinical, and industry sectors, who evaluated the relevance of each item using a 4-point Likert scale (strongly agree, agree, disagree, strongly disagree). Two established quantitative approaches were used to evaluate content validity: (i) content validity ratio (CVR), calculated following Lawshe’s method, where a CVR of ≥0.99 was required for item retention with seven experts at α = 0.05 (Lawshe, 1975); and (ii) content validity index at the item level (I-CVI), the most widely reported content validity in instrument development, where an I-CVI >0.79 indicated item appropriateness, values between 0.70 and 0.79 warranted revision, and values <0.70 necessitated item removal (Abdollahpour et al., 2011; Polit et al., 2007).

Following content validity assessment, 6 items were removed, 10 were revised, and 19 were retained unchanged from the initial 35-item draft. Based on recommendations from seven experts, 13 additional items were incorporated, resulting in a final questionnaire of 41 items across four domains, in addition to a screening question. The questionnaire was then pre-tested with 15 international pharmacometrics experts to evaluate the design, readability, and clarity of the questionnaire. Minor revisions were made, and all participants confirmed that the final version was clear, comprehensible, and easy to complete.

The final survey started with a screening question to confirm eligibility based on clinical experience: “Do you have experience in providing direct patient care?” Respondents selecting “No” were exited from the survey. The four domains included: (i) Demographic, professional, and practice characteristics (10 items); (ii) Awareness and use of Bayesian dose prediction software (5 items); (iii) Attitudes toward the use and implementation of Bayesian dose prediction software (pre-defined list of 13 items); and (iv) Perceived challenges (barriers) to implementation of Bayesian dose prediction software in clinical practice (pre-defined list of 13 items). Items in the attitudes and barriers domains were measured on a 5-point Likert scale (strongly disagree to strongly agree).

### 2.3 Data collection

The questionnaire was electronically distributed to the heads of the eight aforementioned organizations, who were requested to disseminate it to their respective members. Participation was voluntary and anonymous, and informed consent was obtained electronically. To encourage participation, follow-up reminders were sent at 2–3-week intervals to the heads of the organizations.

### 2.4 Statistical analysis

Data were analyzed using IBM SPSS Statistics (version 29.0; IBM Corp., Armonk, NY, United States). Descriptive statistics are reported as percentages (95% confidence intervals [CI]), means (standard deviation [SD]), or medians (interquartile range [IQR]), as appropriate. Normality of attitude and barrier scores was assessed using the Shapiro-Wilk and Kolmogorov-Smirnov tests, confirming non-normal distributions. Consequently, non-parametric tests were applied: the Mann-Whitney test for comparisons between two groups and the Kruskal-Wallis test for comparisons among three or more groups. When significant group differences were detected using the Kruskal-Wallis test, pairwise comparisons were performed using the Dunn-Bonferroni method. Bonferroni-adjusted p-values were reported, with statistical significance set at *p* < 0.05.

Internal consistency of the attitudes and barriers items was assessed using Cronbach’s alpha. The 13 items in the domain of the attitude showed high reliability (α = 0.89), as did the 13 items in the perceived barriers domain (α = 0.83). Scores for attitudes and barriers were categorized as follows based on the overall median values: attitudes (negative [≤3] or positive [≥4]) and barriers (low perceived challenges [≤2] or high perceived challenges [≥3]). One negatively worded item (“The software user is end-responsible for the advice, and there is no need for regulatory body approval for using the software”) in the domain of the attitude was reverse-coded to ensure that higher scores consistently reflected more positive attitudes.

Univariable logistic regression was first conducted to assess the association between independent variables and binary outcomes. Variables with *p* < 0.25 were subsequently included in multivariable logistic regression models to account for potential confounding effects and improve model accuracy, in accordance with established recommendations (Bursac et al., 2008; Mickey and Greenland, 1989). Only results from multivariable models showing significant associations are reported as adjusted odds ratios (AOR) with corresponding 95% CI. Statistical significance was defined as *p* < 0.05.

To ensure an adequate sample size per category for meaningful logistic regression and non-parametric comparison analyses, the included independent variables were categorized as follows: (i) Region (North America, Europe, Middle East, and Others [Africa, Asia, South America, and Australia/New Zealand]); (ii) Educational level (undergraduate degrees [BSc. Pharm, MPharm, and MBBS], professional doctorate/residency degrees [PharmD, MD, and PGY2], and postgraduate degrees [MSc and PhD]); (iii) Current position (pharmacist, physician, and academia/others [research medical Officer, pharmacologist, industry]); (iv) Practice experience since graduation (≤5 years, 6–10 years, 11–20 years, and >20 years); (v) Practice setting (Academic/Teaching hospitals vs. Non-academic hospitals/Infectious disease clinics); (vi) Primary clinical practice setting for spending most of the clinical time (Critical care [adult/pediatric/neonatal] vs. Non-critical care), (vii) TDM practice experience (≤5 years, 6–10 years, 11–20 years, and >20 years); and (viii) Bayesian software use duration (≤5 years, 6–10 years, and >10 years). The three binary dependent variables were: (i) Awareness of Bayesian dose prediction software (Yes = 1, No = 0); (ii) Use of Bayesian dose prediction software in practice (Yes = 1, No = 0); and (iii) Type of Software Used (Moderately/Commonly used [InsightRx, DoseMe, and MWPharm] = 1; Less commonly used = 0).

## 3 Results

### 3.1 Study sample

A total of 234 participants responded to the survey. Of these, 44 respondents (18.8%) indicated that they were not involved in direct patient care and were therefore excluded from completing the survey, which resulted in a final analytical sample of 190 respondents, representing 52.8% of the calculated minimum sample size (360).

### 3.2 Demographic, professional, and practice characteristics of respondents

Table 1 presents the demographic, professional, and practice characteristics of the respondents. Most respondents were based in North America, held a professional doctorate/residency degree as their highest educational qualification, and were pharmacists. Most respondents practiced in academic or teaching hospitals and spent most of their clinical time in non-critical care settings. Engagement in TDM activities was high: 96.8% participated in dose adjustments, 92.6% in initial dosing decisions, 92.6% in interpreting TDM levels, and 87.4% in determining timing for medication concentrations. Most participants had either ≤5 years (30.5%) or 11–20 years (28.4%) of TDM experience, with 61.5% performing TDM-based clinical decisions 2–20 times per week. The top five medications commonly subjected to TDM were vancomycin (92.6%; 95% CI: 87.7–95.8), aminoglycosides (88.4; 95% CI:82.8–92.4), voriconazole (51.6%; 95% CI: 44.3–58.8), tacrolimus (46.8%; 95% CI:39.6–54.2), and phenytoin (46.3%; 95% CI: 39.1–53.6) (Table 2).

**TABLE 1:** Demographic, professional, and practice characteristics of respondents (n = 190).

Characteristics	Frequency (percentage)
Region	
North America	128 (67.4)
Europe	21 (11.0)
Middle East	18 (9.5)
Africa	10 (5.3)
Australia and New Zealand	7 (3.7)
South America	4 (2.1)
Asia	2 (1.1)
Educational level	
Undergraduate degrees [BSc. Pharm, MPharm, MBBS]	6 (3.16)
Professional doctorate/residency degrees [PharmD, MD, PGY2]	128 (67.4)
Postgraduate degrees holders [MSC, PhD]	56 (29.5)
Current professional position	
Pharmacists	146 (76.8)
Physicians	15 (7.9)
Academics-others (research medical officer, pharmacologist, industry)	29 (15.3)
Practice experience since graduation (Years)	
<5 years	44 (23.2)
6–10	43 (22.6)
11–20	55 (28.9)
>20	48 (25.3)
Practice setting	
Academic/Teaching hospital	149 (78.4)
Non-academic hospital/Infectious disease clinic	41 (21.6)
Primary clinical practice setting for the majority of their clinical time	
Critical care	67 (35.3)
Non-critical care	123 (64.7)
Roles in the provision of medicines to hospitalized patients*	
Recommendations/decisions about initial dosing	176 (92.6)
Recommendations decisions about dose adjustments	184 (96.8)
Write orders/prescribe medications	76 (40.0%)
Order/determine timing of medication concentrations (i.e., TDM levels)	166 (87.4)
Interpret medication concentration measurements (i.e., TDM levels)	176 (92.6)
Advice in cases of poisoning	64 (33.7)
Drug level interpretations in cases of poisonings	65 (34.2)
TDM experience	
<5 years	58 (30.5)
6–10	47 (24.7)
11–20	54 (28.4)
>20	31 (16.3)
Average frequency of involvement in TDM-based clinical decisions	
<1 per month	8 (4.2)
1–4 per month	23 (12.1)
2–5 per week	39 (20.5)
5–10 per week	39 (20.5)
10–20 per week	39 (20.5)
20–50 per week	25 (13.2)
>50 per week	17 (8.9)

*Total percentage exceeds 100% due to multiple responses allowed.

**TABLE 2:** Medications Monitored via TDM Programs and Those Managed with Bayesian Dose Prediction Software (*Responses are ordered by descending percentage of medications for which Bayesian software is used*).

Medication	Therapeutic drug monitoring program (n = 190)*	Use of Bayesian dose prediction (n = 102)*
Vancomycin	176 (92.6%)	96 (94.1%)
Aminoglycosides	168 (88.4%)	51 (50%)
Tacrolimus	89 (46.8%)	11 (10.8%)
Methotrexate	60 (31.6%)	10 (9.8%)
Voriconazole	98 (51.6%)	10 (9.8%)
Cefepime	24 (12.6%)	7 (6.9%)
Piperacillin-tazobactam	26 (13.7%)	6 (5.9%)
Meropenem	31 (16.3%)	5 (4.9%)
Mycophenolate	41 (21.6%)	5 (4.9%)
Phenytoin	88 (46.3%)	5 (4.9%)
Fluconazole	28 (14.7%)	4 (3.9%)
Linezolid	25 (13.2%)	4 (3.9%)
Rifampicin	21 (11.1%)	4 (3.9%)
Digoxin	83 (43.7%)	3 (2.9%)
Cefotaxime	10 (5.3%)	2 (2.0%)
Posaconazole	63 (33.2%)	2 (2.0%)
Sirolimus	54 (28.4%)	2 (2.0%)
Teicoplanin	11 (5.8%)	2 (2.0%)
Antipsychotic	34 (17.9%)	1 (1.0%)
Carbamazepine	51 (26.8%)	1 (1.0%)
Ceftazidime	15 (7.9%)	1 (1.0%)
Everolimus	32 (16.8%)	1 (1.0%)
Imipenem - cilastatin	12 (6.3%)	1 (1.0%)
Infliximab	17 (8.9%)	1 (1.0%)
Itraconazole	43 (22.6%)	1 (1.0%)
Pyrazinamide	9 (4.7%)	1 (1.0%)
Tricyclic antidepressants	21 (11.1%)	1 (1.0%)
Amiodarone	21 (11.1%)	0
Azathioprine	24 (12.6%)	0
Cloxacillin	8 (4.2%)	0
Colistin	16 (8.4%)	0
Valproic acid	78 (41.1%)	0

*Total percentage exceeds 100% due to multiple responses allowed.

### 3.3 Awareness and use of Bayesian dose prediction software

A high proportion of respondents reported awareness of Bayesian dose prediction software (n = 157; 82.6%; 95% CI: 76.3–87.6). Of these, 102 (65.0%; 95% CI: 56.9–72.3) reported using Bayesian dose prediction software in clinical practice. The most commonly used Bayesian dosing software were InsightRx (40.2%), DoseMe (22.5%), and MWPharm (18.6%) (Table 3). Among users of Bayesian software, the majority were early users with ≤5 years of experience (n = 74; 72.5%), while 12.7% had 6–10 years and 14.7% had >10 years of experience. The top five medications for which Bayesian software was applied were vancomycin (94.1%; 95% CI: 87.1–97.6), aminoglycosides (50%; 95% CI: 40.0–60.0), tacrolimus (10.8%; 95% CI: 5.8–18.9), methotrexate (9.8%; 95% CI: 5.1–17.7), and voriconazole (9.8%; 95% CI: 5.1–17.7) (Table 2).

**TABLE 3:** Names of Bayesian dosing software used by respondents (*Responses are ordered by descending percentages*) (n = 102).

Software	Frequency (percentage; 95% CI)
InsightRx	41 (40.2; 30.8–50.4)
DoseMe	23 (22.6; 15.1–32.1)
MWPharm	19 (18.6; 11.9–27.8)
TDMx	11 (10.8; 5.8–18.9)
PrecisePK	9 (8.8; 4.4–16.5)
BestDose	6 (5.9; 2.4–12.9)
NextDose	6 (5.9; 2.4–12.9)
Clincalc	4 (3.9; 1.3–10.3)
Mapbayr	2 (2.0; 0.3–7.6)

### 3.4 Attitudes toward the use and implementation of Bayesian dose prediction software

The overall median (IQR) attitude score was 4.0 (2.0), indicating a positive attitude. As shown in Table 4, over 90% of respondents either agreed or strongly agreed that Bayesian software is a valuable clinical decision-support tool, enhances medication safety, and that they understand how Bayesian dosing recommendations are derived. Just over half of the respondents also agreed or strongly agreed that the software user bears end-responsibility for the recommendations, that regulatory body approval is not required for using the software, and that Bayesian dose prediction software is cost-effective.

**TABLE 4:** Attitudes toward the use and implementation of Bayesian dose prediction software (*Responses are ordered by descending percentages*) (n = 102).

Statement	Strongly agreed/Agreed (%)	Mean* (SD)	Median* (IQR)
Bayesian dose prediction software is a useful clinical decision support tool	94 (92.2%)	4.37 (0.73)	4.0 (1.0)
I understand how the Bayesian dosing recommendation was derived	94 (92.2%)	4.27 (0.73)	4.0 (1.0)
Bayesian dose prediction software improves the safety of medications (i.e., reduces toxicity)	93 (91.2%)	4.41 (0.75)	5.0 (1.0)
Bayesian dose prediction software improves patient outcomes	90 (88.2%)	4.18 (0.78)	4.0 (1.0)
Bayesian dose prediction software should be used in daily practice	87 (85.3%)	4.21 (0.88)	4.0 (1.0)
I am confident in the dosing recommendation received from the Bayesian dose prediction software	85 (83.3%)	4.10 (0.80)	4.0 (1.0)
Bayesian dose prediction software improves the efficacy of medications	84 (82.4%)	4.02 (0.83)	4.0 (1.0)
I prefer to use Bayesian dose prediction software that operates in the hospital system to increase patient information confidentiality and privacy	74 (72.5%)	3.95 (0.93)	4.0 (2.0)
The software is easy to use, and output/calculation results are straightforward	65 (63.7%)	3.67 (1.03)	4.0 (1.0)
Bayesian dose prediction software is time-saving	64 (62.7%)	3.67 (1.18)	4.0 (2.0)
There is a need for masterclasses to learn the software	62 (60.8%)	3.58 (1.09)	4.0 (1.0)
The software user is end-responsible for the advice, and there is no need for regulatory body approval for using the software^#^	58 (56.9%)	2.56 (1.10)	2.0 (1.0)
Bayesian dose prediction software is cost-effective	56 (54.9%)	3.65 (0.90)	4.0 (1.0)
Overall attitude	​	3.89 (1.02)	4.0 (2.0)

*Responses rated on a Likert scale ranging from 1 = strongly disagree to 5 = strongly agree.

^#^Negatively worded item was reverse-coded for the score.

### 3.5 Perceived challenges to implementing Bayesian dose software in clinical practice

The median (IQR) score for perceived implementation challenges was 3.0 (2.0), indicating a high level of perceived challenges (Table 5). The top five challenges reported were: (i) the cost to procure the software license was prohibitive; (ii) lack of support from the administration of the health facility for software use and maintenance; (iii) lack of awareness of how to use Bayesian dose prediction software in clinical practice; (iv) the software lacks capability for integration into electronic health records (EHRs); and (v) specialist training required to use Bayesian dose prediction software.

**TABLE 5:** Challenges to implementation of Bayesian dose prediction software in clinical practice (*Responses are ordered by descending percentages*) (n = 157).

Statement	Strongly agreed/Agreed (%)	Mean* (SD)	Median* (IQR)
The cost to get a software license is prohibitive	102 (65.0%)	3.68 (1.01)	4 (2)
There is a lack of support from the administration of the health facility for the use and maintenance of the software	79 (50.3%)	3.41 (1.21)	4 (2)
Lack of awareness of how to use Bayesian dose prediction software in clinical practice	78 (49.7%)	3.23 (1.25)	3 (2)
The software lacks the capability for integration into electronic health records	78 (49.7%)	3.22 (1.29)	3 (2)
Using Bayesian dose prediction software requires specialist training	77 (49.0%)	3.24 (1.17)	3 (2)
Lack of availability of training courses	52 (33.1%)	2.99 (1.06)	3 (2)
I am concerned about the clinical consequences of errors in data entry or interpretation of concentration measurements	50 (31.8%)	2.85 (1.12)	3 (2)
Difficult to implement into the clinical workflow because such software is too time-consuming	46 (29.3%)	2.70 (1.08)	2 (2)
There are several tools, and I am not sure which one to use	45 (28.7%)	2.73 (1.15)	3 (2)
I am concerned about patient data privacy and security when entering the information into the software	32 (20.4%)	2.55 (1.14)	2 (1)
The software is not currently authorized by a regulatory body such as the FDA, so I am not comfortable using it in clinical practice	25 (15.9%)	2.40 (1.02)	2 (1)
Bayesian dose prediction software output is too difficult to understand	15 (9.6%)	2.18 (0.86)	2 (1)
Bayesian dose prediction software is too complicated to use	13 (8.3%)	2.18 (0.84)	2 (1)
• Overall barriers	​	2.82 (1.15)	3.0 (2.0)

*Responses rated on a Likert scale ranging from 1 = strongly disagree to 5 = strongly agree.

### 3.6 Multivariable logistic regression, non-parametric comparison, and correlation results

Multivariable logistic regression analysis revealed that region was the only significant independent variable associated with awareness. Participants from the Middle East had significantly lower awareness compared with those from other regions (AOR = 0.14, 95% CI: 0.05–0.42; p = 0.002). No significant associations were observed between the use of Bayesian software and any independent variables (p > 0.05). However, the current position was identified as the only independent variable significantly associated with the type of software used. Pharmacists were more likely than physicians and other professionals to use moderately/commonly used tools (DoseMe, InsightRx, and MWPharm) (AOR = 7.2, 95% CI: 1.4–17.9; p = 0.003). Long-term users (≥10 years) had significantly more positive attitudes (mean rank = 67.0) than early users (≤5 years) (mean rank = 46.37; adjusted p = 0.021). No significant differences were detected between long-term and intermediate users (6–10 years; mean rank = 60.77; adjusted p = 1.0) or between intermediate and early users (adjusted p = 0.235).

Perceived implementation challenges varied significantly by region and current position. Participants from the Middle East reported higher barrier scores than those from North America (mean rank: 118.13 vs. 75.28; adjusted p = 0.015), while no significant differences were observed between other regions (adjusted p > 0.05). Academics/other professionals reported higher barrier scores than pharmacists (mean rank = 109.0 vs. 71.03; adjusted p < 0.001). No significant differences were observed between pharmacists and physicians (mean rank = 98.0; adjusted p = 1.0), or between physicians and academics/other professionals (adjusted p = 0.091). A negative but non-significant correlation was observed between overall attitude scores and perceived barriers (Spearman’s r = −0.49; p = 0.072), suggesting a trend in which fewer perceived barriers may be associated with more positive attitudes.

## 4 Discussion

To the best of our knowledge, this study represents the first international, multi-organizational survey to explore clinicians’ awareness, usage, attitudes, and perceived barriers toward Bayesian dose prediction software in clinical practice. Its novelty lies in the breadth of participants across different regions, professions, and levels of clinical experience, as well as its endorsement by major pharmacometrics societies. While the clinical utility of Bayesian software has been increasingly demonstrated in the literature, its real-world implementation remains understudied, particularly from the perspective of clinicians who are the end-users of such tools. The current findings provide valuable insights to bridge this gap and guide targeted strategies for promoting broader adoption of precision dosing practices.

The results of this study demonstrate that, despite high awareness and positive attitudes, the clinical use of Bayesian dosing software remains limited, primarily focused on vancomycin and aminoglycosides. Despite belonging to different WHO AWaRe categories, vancomycin and aminoglycosides remain the most common focus of Bayesian dosing because they share characteristics, such as narrow therapeutic indices, high pharmacokinetic variability, and well-defined therapeutic targets that make them particularly suitable for model-informed precision dosing. The top three medications managed with Bayesian dose prediction software, vancomycin, aminoglycosides, and voriconazole, align with those most frequently monitored via TDM. In contrast, phenytoin and digoxin, despite frequent TDM monitoring, showed minimal Bayesian software utilization, likely reflecting the complexity of their pharmacokinetics. This pattern aligns with previous studies (Kantasiripitak et al., 2020; Jager et al., 2022) and may also reflect the lack of adoption of TDM and Bayesian approaches for certain drug classes, such as beta-lactams, where use is often restricted to specific scenarios (e.g., critically ill patients with PK/PD derangements, elevated MICs, or heightened risk of adverse effects). The timing of usage, predominantly within the past 1–5 years, likely reflects the influence of updated consensus guidelines for vancomycin TDM, which encourage area under the curve-based dosing facilitated by Bayesian tools (Rybak et al., 2020). Adoption may also be influenced by the fact that most widely used Bayesian software programs are developed by commercial entities, which often provide stronger user support, regulatory alignment, and marketing compared to academic or in-house solutions. While cost emerged as the most frequently reported barrier in our sample, it is important to recognize that this may not reflect the situation in all regions. In some parts of Asia, for example, groups have developed their own dosing tools or adopted flexible, web-based platforms that can be implemented at a lower cost than commercially licensed software. These local solutions may follow different adoption pathways and may be shaped by health-system priorities that differ from those in predominantly Western settings. Because respondents from Asian countries were underrepresented in our study, these alternative approaches, including those influenced by local stewardship initiatives, institutional policies, or ongoing pharmacometrics activities, are unlikely to be fully reflected in our findings. These findings underscore that expansion of Bayesian dosing practices should be targeted rather than universal, focusing on agents and patient populations where individualized dosing provides the greatest clinical benefit. The most frequently reported barriers to implementing Bayesian dose prediction software were the high cost of licensing, followed by limited institutional support, lack of awareness regarding software use, insufficient integration with EHR systems, and the need for specialist training. Evidence supporting the cost-effectiveness of precision dosing strategies, particularly when combined with therapeutic drug monitoring has begun to emerge, with recent analyses demonstrating potential cost savings and improved clinical outcomes in selected therapeutic areas (Lechug et al., 2025). However, formal economic evaluations specifically addressing commercial Bayesian dose-prediction software remain limited, underscoring the need for further research in this area. Healthcare institutions must consider not only licensing fees but also account for a broader spectrum of associated costs. These include investments in user training, the integration of software into existing EHR systems, and the infrastructure needed to house dosing software, along with the substantial data generated by dosing simulations (Chai et al., 2020). Although many of these costs are difficult to quantify, they can be perceived as substantial barriers by institutional leadership.

The current findings are consistent with earlier literature, which identifies integration and training challenges as key barriers to widespread adoption (Darwich et al., 2017; Chai et al., 2020; Vinks et al., 2020). Although participants reported that the software lacks integration capabilities with EHR systems, a survey of software developers indicated that most Bayesian dosing tools are accessible either through an installed application within Windows-based operating systems or a web-based application that does not require the installation of any software (Mickey and Greenland, 1989). Notably, four programs currently support integration with EHR systems such as Cerner and Epic, which are DoseMe, InsightRX, MwPharm, and PrecisePk. Licensing costs remain highly variable across developers, with tools offering advanced features, including EHR integration, commanding substantially higher fees. Regulatory classification also varies: four software products are registered as medical devices with European regulatory bodies, and one holds dual registration with both European and Australian health authorities (Jager et al., 2022). These differences underscore the need for standardized evaluation frameworks to guide institutional decision-making regarding software selection and implementation.

Although most respondents reported understanding how Bayesian dosing recommendations are derived (92.2%) and expressed high confidence in these recommendations (83.3%), nearly half (49.0%) agreed that using Bayesian dose prediction software requires specialist training, and one-third (33.1%) cited the lack of availability of training courses as a barrier. These seemingly conflicting findings suggest that although many clinicians perceive the software as generally easy to use, structured training remains essential, particularly during initial implementation and when managing complex patient populations. This may reflect clinicians’ recognition that while basic navigation is straightforward, optimal use of model-based outputs requires specialized knowledge and confidence, especially among those less familiar with model-based approaches (Darwich et al., 2017; Chai et al., 2020). Successful implementation of Bayesian dose prediction extends beyond its availability or user familiarity with the interface, critically depending on clinicians’ ability to accurately interpret and apply the PK-PD outputs for individualized therapy in clinical practice. As highlighted in previous literature (Darwich et al., 2017; Chai et al., 2020; Vinks et al., 2020), many clinicians lack the necessary training in pharmacometrics and PK-PD concepts, which can undermine the clinical utility and acceptance of these tools. Without a solid understanding of the principles underlying model-informed precision dosing, even well-designed software may be underutilized or misapplied. Inadequate user education can limit the perceived utility and trust in software-generated recommendations (Darwich et al., 2017). This gap is particularly pronounced in special populations such as the critically ill, where dosing decisions are more complex, and the consequences of error can be more severe (Chai et al., 2020). To address these knowledge and training gaps, structured educational interventions are needed, including targeted training programs, certification pathways, practical workshops focused on Bayesian methods and software use, and integration into postgraduate curricula. Moreover, integrating clinical pharmacists, clinical pharmacologists, or pharmacometric specialists into multidisciplinary care teams could enhance institutional capacity, provide real-time expertise, and foster a culture of evidence-based dosing (Darwich et al., 2017; Chai et al., 2020; Vinks et al., 2020). Such strategies would not only encourage responsible use of Bayesian tools but also accelerate their integration to support evidence-based precision dosing practices.

Other barriers reported in the literature include the absence of high-level PK and technical expertise at practice sites, lack of easy‐to‐use software programs, and challenges in validating licensed programs in the clinical setting (Darwich et al., 2017). Beyond these operational barriers, important scientific considerations also influence the effective use of Bayesian dose-prediction software—particularly in patient groups with substantial PK/PD variability, such as the critically ill or those receiving renal replacement therapies, where expert oversight is often essential (Keizer et al., 2018). Notably, while the commercial availability of Bayesian tools may facilitate adoption, the associated licensing costs can paradoxically serve as a major barrier for many institutions, a finding strongly reflected in our survey results.

The findings of this study reveal that 56.9% of respondents agreed or strongly agreed with the statement that the software user is responsible for the advice and that regulatory body approval is not required for using the software. This finding suggests that while a slight majority perceive regulatory approval as unnecessary, concerns about liability and safety remain relevant. These programs are typically categorized as low-risk medical devices and, as such, are often exempt from rigorous regulatory scrutiny, relying on self-certification of compliance by developers without rigorous demonstration of clinical efficacy or safety (Kantasiripitak et al., 2020; Jager et al., 2022; US Food and Drug Administration, 2022). This regulatory gap has raised concerns among clinicians and healthcare institutions regarding clinical accuracy, safety, and liability of employing such tools in routine care. Jager et al. (2022) and Vinks et al. (2020) have emphasized the need for regulatory authorities to establish a comprehensive, standardized framework for evaluating Bayesian dose prediction software as its adoption continues to expand. Standardized evaluation frameworks for Bayesian dose-prediction tools should, at minimum, include evidence supporting model selection and performance, the ability to integrate with EHR systems, alignment with the expertise level of intended end-users, and provisions for structured training and ongoing support to ensure safe and effective implementation.

### 4.1 Strengths and limitations

This study represents one of the first comprehensive, multi-organizational investigations into clinicians’ awareness, use, attitudes, and perceived barriers regarding Bayesian dose prediction software in real-world clinical settings. By recruiting respondents from diverse professional organizations and geographic regions, the findings may be applicable across a range of healthcare systems. Methodologically, the study employed a rigorously developed and validated questionnaire and applied appropriate statistical analyses, including multivariable logistic regression and non-parametric comparisons to identify factors influencing awareness, usage, attitudes, and perceived challenges. Importantly, the study captured both perceived value and practical implementation barriers, providing a balanced and evidence-based approach to inform future strategies for integrating Bayesian tools into precision dosing practices.

Nevertheless, several limitations must be acknowledged. Most notably, the final sample size (n = 190) did not reach the predetermined target of 360, despite outreach efforts and multiple follow-up reminders coordinated through the leadership of eight professional organizations. This under-enrollment may have reduced the statistical power to detect smaller effect sizes and could have introduced potential response bias if non-respondents held systematically different views. The non-significant correlation between attitude and barrier scores, despite showing a moderate negative trend, should be interpreted cautiously. Given that the achieved sample size was smaller than originally intended, the study may have had limited statistical power to detect associations of this magnitude. Therefore, the lack of statistical significance may reflect insufficient power rather than the absence of a true association. A cross-sectional design restricts the ability to examine changes in awareness, use, attitudes, and barriers over time. Self-reported data may be subject to recall or social desirability biases, despite efforts to maintain respondent anonymity; these inherent biases cannot be entirely ruled out. Although participants were drawn from multiple countries and professional backgrounds, the sample was dominated by respondents from North America, with very limited input from Asia, South America, and Africa. This imbalance restricts the extent to which our results can be applied globally. Patterns of Bayesian dosing tool adoption are strongly shaped by local clinical practices, technological capacity, organizational priorities, and the maturity of digital health systems, all of which vary substantially between regions. In particular, many Asian health systems differ markedly in terms of therapeutic drug monitoring infrastructure, institutional workflows, procurement processes, and readiness for advanced clinical decision support. Because these settings were only minimally represented in our sample, important variation in how Bayesian approaches are introduced and integrated into practice may not be reflected in our findings. Future research should actively involve a broader geographical distribution of respondents to better characterize the range of implementation strategies and contextual influences across diverse healthcare environments. Future work will be complemented by regional expert perspectives particularly from Asia to contextualize adoption pathways, policy environments, and locally developed software ecosystems that were underrepresented in the present sample. Because the survey was distributed through professional organizations rather than individual institutions, we were not able to determine whether multiple participants came from the same hospital or health system. Without institutional identifiers, it was not possible to assess or adjust for potential clustering of responses. As a result, the perspectives of institutions with more active members may have been more represented in the dataset, which should be considered when interpreting the findings. Future research may benefit from qualitative approaches, such as focus group discussions or in-depth interviews, to explore institutional contexts and workflow considerations that differ across tertiary hospitals, secondary hospitals, and primary care settings. Furthermore, the exclusion of respondents not directly involved in patient care, while appropriate for the clinical focus of the study, may have omitted valuable perspectives from key stakeholders such as administrators, clinical informatics specialists, or decision-makers who often play a pivotal role in the implementation and institutional adoption of clinical decision-support tools. Aminoglycosides were treated as a class, and information regarding the availability of individual agents (e.g., amikacin vs. gentamicin vs. tobramycin) for the TDM program or the use of Bayesian dose prediction software was not collected. Finally, the present survey focused on the established use of Bayesian tools for dose individualization of conventional TDM-guided medications. Broader applications, such as adherence-informed interpretation and individualized longitudinal treatment assessment, were beyond the scope of this work and should be explored in future research. These limitations underscore the need for future longitudinal research with broader stakeholder inclusion and more representative sampling. Despite these limitations, the study provides timely, real-world insights into an underexplored but increasingly relevant area of clinical decision support. It offers a foundational understanding of the current landscape of Bayesian software adoption and highlights key areas for policy, educational, and regulatory focus to support broader implementation in precision pharmacotherapy.

## 5 Conclusion

This global survey provides novel and comprehensive insights into clinicians’ awareness, use, attitudes, and perceived barriers toward Bayesian dose prediction software in clinical practice. Despite high awareness and generally positive attitudes toward Bayesian dosing software, its clinical use remains limited, primarily focused on vancomycin and aminoglycosides, and largely restricted to a small number of commercially supported tools. Targeted interventions are needed to address key implementation barriers. Notable differences were observed across regions and professional roles, particularly in awareness and perceived implementation challenges, underscoring the need for context-specific strategies to improve implementation. The identification of cost, lack of institutional support, lack of awareness of how to use Bayesian dose prediction software in clinical practice, limited integration with EHRs, and the need for specialist training as key barriers reflect systemic and infrastructural gaps that must be addressed for broader implementation. While advanced tools such as Bayesian software hold considerable potential to individualize dosing and improve patient outcomes, their successful adoption requires regulatory clarity, dedicated funding, education, and seamless integration into clinical workflows. Notably, the observed disconnect between awareness and use, as well as between attitudes and perceived barriers, underscores the complexity of digital health adoption. Future efforts should focus not only on technical validation and regulatory oversight but also on interprofessional training and qualitative research to better understand user needs, workflow integration, and institutional readiness. As precision dosing continues to evolve, addressing these multifaceted challenges will be essential for realizing the full potential of model-informed precision dosing in routine clinical practice.

## Data Availability

The raw data supporting the conclusions of this article will be made available by the authors, without undue reservation.
